# Mental Disorders and Having a First Child Among Young Adults: A Nationwide Register‐Based Cohort Study

**DOI:** 10.1111/1471-0528.18151

**Published:** 2025-03-24

**Authors:** Kateryna Golovina, Ripsa Niemi, Mai Gutvilig, Markus Jokela, Marko Elovainio, Christian Hakulinen

**Affiliations:** ^1^ Department of Psychology, Faculty of Medicine University of Helsinki Helsinki Finland; ^2^ Population Research Institute Väestöliitto–Family Federation of Finland Helsinki Finland; ^3^ Finnish Institute for Health and Welfare Helsinki Finland

**Keywords:** becoming a parent, fertility, mental disorders, partnership status, register‐based study

## Abstract

**Objective:**

To examine associations between mental disorders and time to first childbirth in Finland, and whether partnership status mediates these associations.

**Design:**

Nationwide register‐based cohort study.

**Setting:**

Primary and secondary healthcare data from Finland.

**Population:**

All individuals born in 1980–1995 who were childless and living in Finland at age 16 (*n* = 1 210 662).

**Methods:**

Cox proportional hazards models to examine associations between mental disorders and time to first childbirth. Participants were followed until first childbirth, death, emigration, or the end of 2019.

**Main Outcome Measures:**

Time to first live childbirth.

**Results:**

Both men and women diagnosed with mental disorders had a lower likelihood of becoming parents compared to those without diagnoses. People diagnosed with schizophrenia and intellectual disabilities were the least likely to become parents. Adjusting for partnership status attenuated the associations for all mental disorders. Before age 25, substance use, childhood onset, anxiety, or any mental disorders were associated with a *higher* likelihood of first childbirth, but after age 30, mental disorders were linked to a *lower* likelihood of parenthood.

**Conclusions:**

Almost all mental disorders were associated with a lower likelihood of having a first child among young people born in 1980–1995. These findings imply that well‐functioning mental health services are important from a fertility perspective.

## 1 Introduction

The postponement of first births has become a significant demographic trend in many high‐income countries [1], contributing to declining fertility rates since the 2010s [2, 3, 4, 5, 6]. Factors driving this delay include educational and career aspirations, economic uncertainty, rising childbearing costs, changing partnership patterns, and shifting social norms [1, 7, 8]. Mental disorders may also play a role, particularly given the increase in mental health problems among young people since 2000 [9, 10, 11, 12], although they are often less studied in demographic research. Antidepressant use, for instance, doubled in OECD countries between 2000 and 2015 [13], and disability pensions due to mental disorders have increased in several countries [14, 15, 16].

Previous register‐based studies found that people with major psychiatric disorders, particularly men, have lower fertility rates compared to the general population [17, 18, 19, 20]. Severe mental health conditions like schizophrenia and bipolar disorder have the strongest associations with reduced fertility [17, 19, 21], though common mental disorders also contribute to lower childbearing rates [17, 19, 22]. Among all early‐life diseases, mental and behavioural disorders had the strongest associations with lifetime childlessness, as recently shown using register data from Finland and Sweden [19]. Although mental disorders may directly influence childbearing through medical conditions that affect fecundity [23], difficulties in forming stable partnerships may potentially explain this association. People with mental disorders are more likely to remain single or experience unstable relationships [24, 25], which may further explain delayed or reduced childbearing.

However, most prior studies focused on older cohorts born between 1950 and 1980, making their findings less applicable to younger generations. Additionally, many studies used only secondary healthcare data, excluding individuals with milder symptoms treated in primary care. This creates a selected sample, potentially leading to conservative estimates. Our study addressed these limitations by examining more recent birth cohorts (1980–1995) and using both primary and secondary healthcare data from Finnish nationwide registers. We first examined how different mental disorders are associated with the time to the first childbirth separately among men and women and by different ages (age 25, 30, and 35). We expected that mental disorders in young people are related to a lower likelihood of having a first child. Second, we also investigated whether partnership status mediates the associations between mental disorders and delayed childbearing, hypothesising that difficulties in forming partnerships account for much of the link between mental health issues and first births.

## 2 Methods

### 2.1 Study Population

All individuals born in 1980–1995 were followed from their 16th birthday until their first live birth, death, emigration from Finland, or end of 2019, whichever occurred first. Individuals who died or emigrated were right censored. The maximum length of follow‐up was 23 years for those born in 1980, reaching a maximum age of 39. Given that there were few individuals who had their first child before age 16 (*n* = 667, 0.13% of individuals who had a first child), they were excluded from the study. The data on mental disorders were retrieved from the Care Register for Health Care (CRHC), the Hospital Discharge Register (HDR), and the Register of Primary Healthcare Visits (RPHV) and linked with data on the birthdate of the parent's first child from the full Finnish Population Register (FOLK) of Statistics Finland. Individual‐level register linkages were conducted using personal identity numbers, which are assigned to all Finnish residents. The ethics committee of the Finnish Institute for Health and Welfare (THL/184/6.02.01/2023§933) approved the study on February 14, 2023. Data were linked with the permission of Statistics Finland (TK‐53‐1696‐16) and the Finnish Institute for Health and Welfare. Informed consent is not required for register‐based studies in Finland.

### 2.2 Measures

#### 2.2.1 Exposure

Mental disorder diagnoses (International Classification of Diseases 10th revision [ICD‐10] F10 to F99 and equivalent ICD‐8, ICD‐9, and International Classification of Primary Care, second edition [ICPC‐2] codes), included inpatient hospital episodes (1970–2019), secondary outpatient visits (1998–2019), and primary healthcare visits (2011–2019) in Finland. In our study, broad categories of mental disorders across the whole ICD‐10 subchapter *F* were used: substance use disorders (F10–F19), psychotic disorders (F20–F29), mood disorders (F30–F39), anxiety disorders (F40–F48), behavioural syndromes associated with physiological disturbances and physical factors (F50–F59), personality disorders (F60–F69), intellectual disabilities (F70–F79), developmental disorders (F80–F89), and childhood onset disorders (F90–F98). We also used several specific categories of mental disorders: schizophrenia (F20), bipolar disorder (F30–F31), depressive disorder (F32–F34), eating disorders (F50) and sleep disorders (F51). Finally, we also examined any mental disorder (F00–F99) as its own category.

#### 2.2.2 Outcome

Time to the birth of the first child was used as an outcome in all analyses.

#### 2.2.3 Control variables

Being married/cohabiting (0 = *never cohabited or married*, 1 = *cohabited or married at least once*) was identified from the population register of Statistics Finland. A cohabiting couple is defined as two unmarried adults of different genders, aged 18 or older, who live together permanently in the same residence. This definition applies if the age difference between the individuals is less than 16 years and they are not siblings [26]. However, if the couple has a common child, these criteria do not apply [26]. Other control variables included year of birth (used as a continuous variable), level of education defined at the end of the follow‐up period (1 = *less than upper secondary*, 2 = *upper secondary*, or 3 = *tertiary*), and diseases of the genitourinary system identified using ICD‐10 codes from the primary and secondary healthcare (1996–2019): male infertility (N46), endometriosis (N80), absent, scanty and rare menstruation (N91) and female infertility (N97). Diseases of the genitourinary system were dichotomised and used as a binary variable in the analyses (0 = *none of these diseases before or during follow‐up*, 1 = *at least one of the diseases before or during follow‐up*).

### 2.3 Statistical Analysis

#### 2.3.1 Main Analysis

We used Cox proportional hazards models to estimate the hazard ratio (HR) of having a first child in parents with a mental disorder diagnosis compared to parents with no mental disorder diagnosis, with men and women analysed separately. Mental disorders were treated as time‐varying variables: persons were classified as unexposed until the date they received a diagnosis and exposed thereafter (0 = *no mental disorder diagnosis*, 1 = *mental disorder diagnosis*). In the present study, if the HRs are lower than 1, the likelihood of having a first child is lower among people with a mental disorder diagnosis than among people without mental disorder diagnoses, and vice versa. Two models were built: the minimally adjusted model included birth year, educational level, and infertility‐related genitourinary diseases as control variables (Model 1), whereas the fully adjusted model additionally included cohabitation/marriage to examine its role in the association (Model 2). We also estimated associations between mental disorders and cohabitation/marriage using modified Poisson regressions adjusting for birth year and education (relative risks are considered more interpretable than odds ratios when the outcome has a prevalence of over 10% [27]). Moreover, to examine associations at different points of the fertility period, we analysed associations between mental disorders and time to the first child by ages 25, 30 and 35. We also studied absolute differences by estimating the cumulative incidence of having a first child for people with and without mental disorder diagnoses using the Aalen‐Johansen estimator, where death and emigration were considered competing events. There was no missingness in the covariates, as data on these covariates from all Finnish residents is systematically recorded in the nationwide registers.

#### 2.3.2 Sensitivity Analysis

To gauge the degree to which associations were confounded by genetics and early environment, we conducted sibling analyses using all individuals who had full siblings in the cohort. Family‐stratified Cox proportional hazards models were used with mental disorders as binary time‐invariant covariates (0 = *no diagnosis before or during follow‐up*, 1 = *diagnosis before or during follow‐up*) and adjusted for birth year, sex, educational level, cohabiting and infertility‐related genitourinary diseases. Men and women were analysed together. Sibling analyses were compared to corresponding Cox proportional hazards models with no family stratification using all individuals in the sample. Finally, we repeated the main analyses examining associations between mental disorders and time to the first child using only secondary health care data.

Statistical analyses were conducted using Stata (version 17.0) and R (version 4.2.2) employing packages *tidyverse* v.1.3.2 and *etm* v.1.1.1 [28, 29].

## 3 Results

Table 1 shows the characteristics of the study population. A total of 1 210 662 individuals (48.5% women) were included in the analyses and followed for 15.4 million person‐years. The median age at first birth was 28 years for men and 26 years for women. During the follow‐up, 36.4% of men and 49% of women had their first child, and the rest did not have a first child. Moreover, 24.2% of men and 28.2% of women received a mental disorder diagnosis prior to or during the follow‐up. Anxiety disorders were the most prevalent in the study population, followed by mood disorders, substance use disorders for men, and behavioural syndromes with physical factors for women (Table S1).

**TABLE 1:** Characteristics of the sample.

Characteristics	Men			Women		
	No mental disorder	Any mental disorder	Total	No mental disorder	Any mental disorder	Total
*N*	472 689	150 860	623 549	421 476	165 637	587 113
Person years	6 274 923	2 046 162	8 321 084	4 978 179	2 084 019	7 062 198
Median follow‐up time (years)^a^	12.9 (9.9, 16.5)	12.9 (10, 16.9)	12.9 (9.9, 16.5)	11.5 (8.6, 15)	12 (9.3, 15.7)	11.7 (8.8, 15.1)
	*N* (%)	*N* (%)	*N* (%)	*N* (%)	*N* (%)	*N* (%)
Had a first child	196 009 (41%)	31 171 (21%)	227 180 (36%)	234 010 (56%)	53 887 (33%)	287 897 (49%)
Emigration	18 291 (3.9%)	1863 (1.2%)	20 154 (3.2%)	18 844 (4.5%)	3345 (2.0%)	22 189 (3.8%)
Death	3185 (0.7%)	3446 (2.3%)	6631 (1.1%)	862 (0.2%)	1358 (0.8%)	2220 (0.4%)
Birth year						
1980–1985	197 543 (42%)	43 894 (29%)	241 437 (39%)	181 240 (43%)	44 086 (27%)	225 326 (38%)
1986–1990	147 768 (31%)	46 457 (31%)	194 225 (31%)	130 191 (31%)	52 561 (32%)	182 752 (31%)
1991–1995	127 378 (27%)	60 509 (40%)	187 887 (30%)	110 045 (26%)	68 990 (42%)	179 035 (30%)
Age at first birth^a^	27.6 (24.5, 30.7)	26.7 (23.6, 30.3)	27.5 (24.3, 30.7)	26.2 (22.7, 29.3)	25.9 (22.5, 29.5)	26.1 (22.7, 29.4)
Genitourinary system diseases						
Has at least one diagnosis	4639 (1.0%)	1157 (0.8%)	5796 (0.9%)	28 238 (6.7%)	17 826 (11%)	46 064 (7.8%)
No diagnosis	468 050 (99%)	149 703 (99%)	617 753 (99%)	393 238 (93%)	147 811 (89%)	541 049 (92%)
Ever cohabitated						
Has cohabitated	323 169 (68%)	83 465 (55%)	406 634 (65%)	319 144 (76%)	121 269 (73%)	440 413 (75%)
Never cohabitated	149 520 (32%)	67 395 (45%)	216 915 (35%)	102 332 (24%)	44 368 (27%)	146 700 (25%)
Education at end of follow‐up						
Lower secondary or less	110 134 (23%)	42 370 (28%)	152 504 (24%)	96 699 (23%)	31 002 (19%)	127 701 (22%)
Upper secondary	226 250 (48%)	83 791 (56%)	310 041 (50%)	154 344 (37%)	79 345 (48%)	233 689 (40%)
Post‐secondary or tertiary	136 305 (29%)	24 699 (16%)	161 004 (26%)	170 433 (40%)	55 290 (33%)	225 723 (38%)

^a^ Median (interquartile range).

**FIGURE 1:**
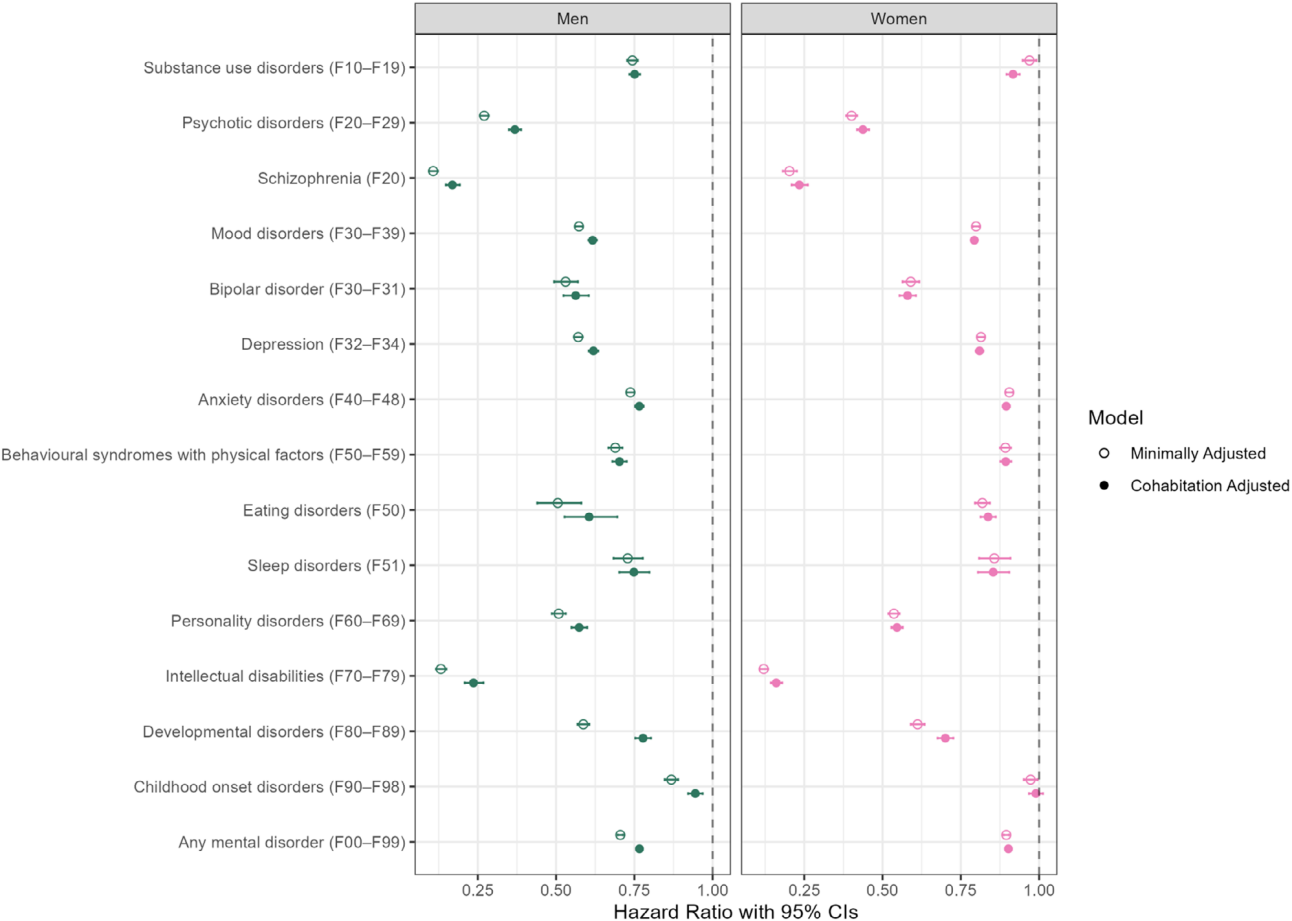
Hazard ratios (95% confidence intervals) of the association between mental disorders and time to the first child. Estimates are shown in Table S2.

Figure 1 shows associations between mental disorders and time to the first child among men and women in the minimally and fully adjusted models (estimates are shown in Table S2). Overall, almost all mental disorders were associated with a lower hazard rate of having a first child in both models. The strongest associations were found for psychotic disorders (HR = 0.26, 95% CI 0.26–0.28 for men and HR = 0.40, 95% CI 0.38–0.42 for women in the minimally adjusted model) and intellectual disabilities (HR = 0.13, 95% CI 0.12–0.15 for men and HR = 0.12, 95% CI 0.11–0.13 for women in the minimally adjusted model). Substance use disorders, mood disorders, anxiety disorders, and sleep and eating disorders were related to a lower hazard rate of having a first child in men compared to women; whereas intellectual disability and developmental disorders were found in women compared to men. For example, men diagnosed with schizophrenia were 83% less likely to have a first child than men without it (77% for women). Among more common disorders, men with mood disorders were 38% less likely to have a first child compared to men without it (21% for women); anxiety disorders—23% for men and 10% for women, and substance use disorders—25% for men and 8% for women.

After adjusting for cohabitation/marriage, the associations attenuated for almost all mental disorders, especially among men (Figure 1, fully‐adjusted model). This suggests that partnership status partially explains the associations between mental disorders and first births. The likelihood of having a partner was lower for people with mental disorders compared to people without them (Figure S1; Table S3). Men diagnosed with schizophrenia were 80% less likely to cohabit than men without it (63% for women). Generally, men's rate ratios of cohabiting were smaller across all mental disorders than those of women's (Figure S1; Table S3).

**FIGURE 2:**
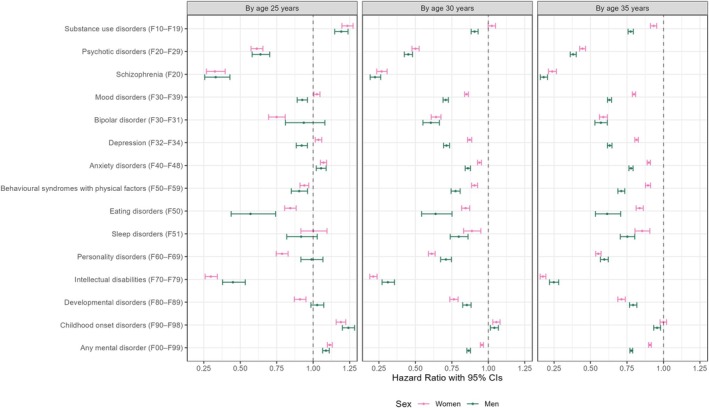
Age‐specific hazard ratios (95% confidence intervals) of the association between mental disorders and time to the first child. Estimates are shown in Table S4.

Figure 2 shows associations between mental disorders and time to the first child by age (estimates in Table S4). Both men and women with substance use (men: HR = 1.19, 95% CI 1.15–1.24; women: HR = 1.23, 95% CI 1.20–1.27), childhood onset (men: HR = 1.24, 95% CI 1.20–1.28; women: HR = 1.19, 95% CI 1.16–1.22), anxiety (men: HR = 1.05, 95% CI 1.02–1.09; women: HR = 1.07, 95% CI 1.05–1.09) or any mental disorders (men: HR = 1.09, 95% CI 1.07–1.11; women: HR = 1.11, 95% CI 1.10–1.13) had a higher hazard rate of having a first child by age 25 compared to those without these disorders. However, by age 30–35, these associations became negative or non‐significant. By age 25, gender differences in these associations were negligible but became more pronounced with age.

**FIGURE 3:**
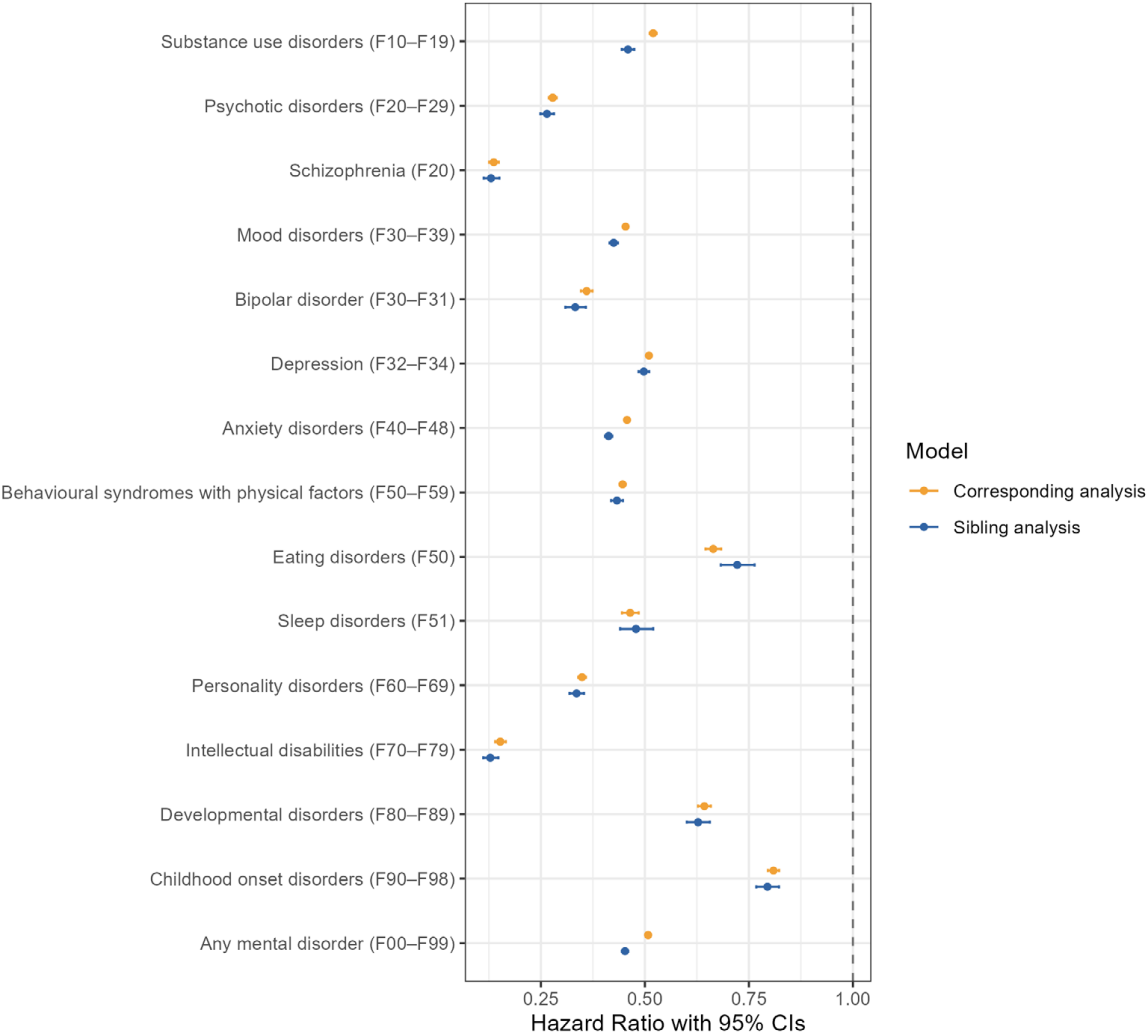
Hazard ratios (95% confidence intervals) of the association between mental disorders and time to the first child of the sibling analysis and corresponding model without family stratification. Estimates are shown in Table S5. Corresponding analysis refers to models with no family stratification using all men and women in the sample.

Sibling analysis showed very similar results to the main analysis (Figure 3; Table S5). Substance use disorders, mood disorders, anxiety disorders, and any mental disorders were associated with a lower hazard rate of having a first child in sibling analyses than in corresponding analyses.

The cumulative incidence of having a first child was lower in those diagnosed with mental disorders (Figure S2; Table S6). For both men and women with developmental disorders, and for women with substance use disorders, there was no change in the cumulative incidence of having a first child based on the exposure before the age of 22 (24 for women with substance use disorders). Up to age 24, individuals with childhood‐onset disorders were more likely to have their first child than those without; however, this association reversed in later years.

### 3.1 Sensitivity Analysis

Analyses fitted with only secondary health care data were very similar to the analyses fitted with both primary and secondary health care data (Figure S3; Table S7).

## 4 Discussion

### 4.1 Main Findings

Drawing on the Finnish population‐based register data, this cohort study showed that young men and women born in 1980–1995 diagnosed with mental disorders prior to parenthood had a lower likelihood of having their first child compared to those without such diagnoses during the follow‐up period. The results from sibling analysis were similar to the main findings, suggesting that they are robust to shared familial factors. People with severe mental disorders were the least likely to become parents. For example, 56% of men in the general population had a child by age 39, but this proportion was only 6% for men diagnosed with schizophrenia. This difference was 69% versus 20% for women. Among the more common mental disorders, mood disorders were strongly associated with a lower likelihood of having a first child: 58% in the general population versus 28% for those diagnosed with mood disorders in men (72% vs. 50% in women). For almost all mental disorders, the associations were stronger for men than women. These findings may be explained by the low likelihood of cohabiting among people with mental disorders, especially among men.

### 4.2 Interpretation

Overall, our findings were in line with previous studies in older birth cohorts showing that men and women with mental disorders have fewer children [17, 19, 20, 22]. Our findings showed that all mental disorders (except childhood onset disorders in women) were associated with a lower likelihood of having a first child both among men and women born in 1980–1995. An important finding was that both men and women with substance use disorder, anxiety, childhood onset disorders, or any mental disorder were more likely to have a first child by age 25, but after that these disorders started to predict a lower likelihood of first births. This accords with a previous study showing that externalising behaviour in childhood both in men and women were associated with an earlier transition to parenthood, but this association attenuated with age [30]. Among women, internalising problems in childhood were related to a higher likelihood of parenthood by age 25, but after that it started to predict a lower fertility rate among women [30].

The associations were stronger for men compared to women, which has been reported in earlier studies as well [17, 19, 20, 22]. This might be related to disease severity and differences in reporting of symptoms. For example, among women, only severe depression or severe depression with psychosis was related to a lower likelihood of having children, whereas for men, even mild depression was associated with having fewer children [22]. Women are more likely to recognise subtle emotional changes and report mild–moderate depressive symptoms, whereas men report more severe cases [31]. Since current diagnostic criteria have equal cut‐off points for both genders, women are more likely to be diagnosed, whereas male depression often remains underdiagnosed [32]. Additionally, moderate depressive symptoms are linked to inflammation in men, but not in women, suggesting depression may have different psychological and physiological implications for men and women [33].

People with mental disorders, men in particular, were less likely to have a partner. For example, men with schizophrenia and intellectual disabilities were 80% less likely to cohabit compared to men without these diagnoses. The challenge of forming and maintaining relationships is influenced by the severity of symptoms, as indicated by our findings, and is consequently linked to a lower likelihood of becoming a parent. Financial strain, often associated with mental disorders [34, 35], may further contribute to difficulties in finding a partner or establishing stable relationships. It should be noted, however, that we did not conduct a formal mediation analysis. Taking this into account, mental health services can help address disparities in partnership formation among people with mental disorders. For example, cognitive‐behavioural therapy can help people develop relationship and communication skills, while peer support groups promote social interactions and may reduce feelings of isolation. Public awareness campaigns can help reduce stigma, encouraging acceptance and confidence in building relationships. Overall, the diagnosis and treatment of mental disorders early in life has been shown to be beneficial for many life outcomes [36] and therefore may also be helpful in terms of partnership formation and the likelihood of having children.

### 4.3 Strengths and Limitations

The strength of our study was the use of both primary and secondary health care data from the Finnish nationwide registers, which allowed us to examine the associations with high statistical precision and minimal health‐related selection biases. Register‐based mental disorder diagnoses are generally shown to have good validity [37]; however, some disorders lack validation, and differences may exist between clinical settings. The birth statistics include data on all liveborn and stillborn children whose mothers were permanently residing in Finland at the time of birth. We also focused on recent birth cohorts (i.e., born in 1980–1995) to examine childbearing patterns among younger people, given that they contribute the most to the ongoing fertility decline in Finland.

However, some limitations should be considered. First, only mental disorders diagnosed in health care settings could be identified in our study. Primary health care data use should encompass people with less severe symptoms, partly representing people with mental disorders who do not seek professional healthcare. However, the absence of data on people with undiagnosed mental disorders may introduce bias, potentially attenuating the observed associations between mental disorders and the likelihood of becoming a parent. Second, since our study relied solely on register data, we were unable to control for fertility preferences or distinguish voluntary from involuntary childlessness. A recent study found that the desire to remain childless has increased significantly among Finns born in 1985–1994 compared to earlier cohorts [38]. While this trend could affect our results, the influence of mental disorders on the desire to remain childless remains unclear. Third, we adjusted the analyses for several control variables to account for the effects of socio‐economic status and health conditions related to fertility on the associations between mental disorders and becoming a parent. Additionally, we examined whether partnership status mediates these associations. However, unmeasured confounders may still influence the relationship between mental disorders and having a first child, even though sibling analyses suggested minimal differences due to shared genetic and early environmental factors. As this is an observational study, causality cannot be established, and reverse causality between mental disorders and partnership status is possible. Finally, younger cohorts included in our study may still have children, and their total fertility period was not covered.

## 5 Conclusions

In conclusion, this study showed a consistent trend across various mental disorders, indicating a reduced likelihood of having a first child among young men and women. Schizophrenia and intellectual disabilities had the strongest associations. People with mental disorders, especially men, were also less likely to cohabit, which could potentially account for the identified patterns. These findings underscore the need for a comprehensive care approach that includes reproductive counselling and support from mental health professionals as part of the treatment plan. Since the impact of mental disorders on parenthood is stronger for men, gender‐specific strategies might be beneficial; in particular, interventions that focus on improving relationship skills could be especially useful for men. Moreover, continuous monitoring and long‐term support for individuals with mental disorders are essential to ensure that they receive appropriate guidance and support in life decisions, including parenthood.

## Author Contributions

**Kateryna Golovina:** conceptualisation; methodology; funding acquisition; roles/writing – original draft; writing – review and editing. **Ripsa Niemi:** conceptualisation; formal analysis; methodology; writing – review and editing; visualisation. **Mai Gutvilig:** conceptualisation; formal analysis; methodology; writing – review and editing; visualisation. **Markus Jokela:** conceptualisation; funding acquisition; writing – review and editing. **Marko Elovainio:** conceptualisation; funding acquisition; writing – review and editing. **Christian Hakulinen:** conceptualisation; data curation; methodology; supervision; funding acquisition; writing – review and editing. All authors contributed to and have approved the final manuscript.

## Ethics Statement

The ethics committee of the Finnish Institute for Health and Welfare (THL/184/6.02.01/2023§933) approved the study on February 14, 2023. Data were linked with the permission of Statistics Finland (TK‐53‐1696‐16) and the Finnish Institute for Health and Welfare. Informed consent is not required for register‐based studies in Finland.

## Conflicts of Interest

The authors declare no conflicts of interest.

## Supporting information


Data S1.

## Data Availability

The data used in this study are the property of Statistics Finland and the Finnish Institute of Health and Welfare. The data are available from the respective authorities, but certain restrictions apply. For more information on accessing the data, please visit www.stat.fi.
