# Supplementary material for: Mental Disorders and Having a First Child Among Young Adults: A Nationwide Register‐Based Cohort Study
Source: BJOG. 2025 Mar 24;132(8):1147–55. doi: 10.1111/1471-0528.18151 (PMC12137792; doi:10.1111/1471-0528.18151)
Supplement: Supplementary file 1 — Data S1. [file BJO-132-1147-s001.docx]

**Mental disorders and having a first child among young adults:**

**A nationwide register-based cohort study**

**Supporting information**

| **Table S1.** | Prevalence of mental disorders in the study population. |
| --- | --- |
| **Table S2.** | Hazard ratios (95% confidence intervals) of the association between mental disorders and having a first child. |
| **Table S3.** | Rate ratios (95% confidence intervals) of the association between mental disorders and cohabitation. |
| **Table S4**. | Age-specific hazard ratios (95% confidence intervals) of the association between mental disorders and having a first child. |
| **Table S5.** | Hazard ratios (95% confidence intervals) of the association between mental disorders and having a first child of the sibling analysis and corresponding model without family stratification. |
| **Table S6.** | Estimates for cumulative incidence of having a first child among men and women with and without mental disorders. |
| **Table S7.** | Hazard ratios (95% confidence intervals) of the association between mental disorders and having a first child using only secondary health care data. |
| **Figure S1.** | Rate ratios (95% confidence intervals) of the association between mental disorders and cohabitation. |
| **Figure S2**. | Cumulative incidence of having a first child among men and women with and without mental disorders. |
| **Figure S3.** | Hazard ratios (95% confidence intervals) of the association between mental disorders and having a first child using only secondary health care data. |

**Table S1.** Prevalence of mental disorders in the study population.

| **Mental disorders** | **Men** | **Women** |
| --- | --- | --- |
| Any mental disorder (F00-F99) | 24.2% (150,860) | 28.2% (165,637) |
| Substance use disorders (F10-F19) | 5.9% (37,046) | 3.6% (21,117) |
| Psychotic disorders (F20-F29) | 2.3% (14,293) | 1.5% (9,076) |
| Mood disorders (F30-F39) | 8.7% (54,199) | 13.4% (78,525) |
| Anxiety disorders (F40-F48) | 11.1% (69,511) | 17.3% (101,405) |
| Behavioral syndromes with physical factors (F50-F59) | 4.3% (27,066) | 6.7% (39,545) |
| Personality disorders (F60-F69) | 1.9% (11,735) | 2.6% (15,435) |
| Intellectual disability (F70-F79) | 0.8% (4,854) | 0.6% (3,500) |
| Developmental disorders (F80-F89) | 4.3% (26,552) | 2.1% (12,304) |
| Childhood onset disorders (F90-F98) | 4.9% (30,270) | 3.8% (22,144) |
| Schizophrenia (F20) | 0.8% (4,786) | 0.4% (2,199) |
| Bipolar disorder (F30-F31) | 0.7% (4,548) | 1.2% (7,146) |
| Depressive disorder (F32-F34) | 6.1% (38,006) | 9.9% (58,408) |
| Eating disorders (F50) | 0.2% (1,335) | 2.5% (14,702) |
| Sleep disorders (F51) | 0.8% (5,115) | 0.7% (4,356) |
| n (persons) | 623549 | 587113 |
| n (person years) | 8321084 | 7062198 |

*Note.* Percentages (numbers) are reported.

**Table S2.** Hazard ratios (95% confidence intervals) of the association between mental disorders and having a first child.

|  | **Men (person years=8321084)** | | **Women (person years = 7062198)** | |
| --- | --- | --- | --- | --- |
|  | Model 1 | Model 2 | Model 1 | Model 2 |
| **Diagnosis** | HR (95% CI) | HR (95% CI) | HR (95% CI) | HR (95% CI) |
| Substance use disorders (F10–F19) | 0.74 (0.73, 0.76) | 0.75 (0.73, 0.77) | 0.97 (0.95, 0.99) | 0.92 (0.90, 0.94) |
| Psychotic disorders (F20–F29) | 0.27 (0.26, 0.28) | 0.37 (0.35, 0.39) | 0.40 (0.38, 0.42) | 0.44 (0.42, 0.46) |
| Schizophrenia (F20) | 0.11 (0.09, 0.12) | 0.17 (0.15, 0.19) | 0.20 (0.18, 0.23) | 0.23 (0.21, 0.26) |
| Mood disorders (F30–F39) | 0.57 (0.56, 0.59) | 0.62 (0.60, 0.63) | 0.80 (0.79, 0.81) | 0.79 (0.78, 0.80) |
| Bipolar disorder (F30–F31) | 0.53 (0.49, 0.57) | 0.56 (0.52, 0.60) | 0.59 (0.56, 0.62) | 0.58 (0.55, 0.61) |
| Depression (F32–F34) | 0.57 (0.56, 0.58) | 0.62 (0.60, 0.63) | 0.81 (0.80, 0.83) | 0.81 (0.80, 0.82) |
| Anxiety disorders (F40–F48) | 0.74 (0.72, 0.75) | 0.77 (0.75, 0.78) | 0.90 (0.89, 0.92) | 0.90 (0.88, 0.91) |
| Behavioural syndromes with physical factors (F50–F59) | 0.69 (0.67, 0.71) | 0.70 (0.68, 0.73) | 0.89 (0.88, 0.91) | 0.89 (0.88, 0.91) |
| Eating disorders (F50) | 0.51 (0.44, 0.58) | 0.61 (0.53, 0.70) | 0.82 (0.80, 0.84) | 0.84 (0.81, 0.86) |
| Sleep disorders (F51) | 0.73 (0.68, 0.78) | 0.75 (0.70, 0.80) | 0.86 (0.81, 0.91) | 0.85 (0.81, 0.90) |
| Personality disorders (F60–F69) | 0.51 (0.49, 0.53) | 0.57 (0.55, 0.60) | 0.54 (0.52, 0.55) | 0.55 (0.53, 0.56) |
| Intellectual disabilities (F70–F79) | 0.13 (0.12, 0.15) | 0.24 (0.21, 0.27) | 0.12 (0.11, 0.13) | 0.16 (0.14, 0.18) |
| Developmental disorders (F80–F89) | 0.59 (0.57, 0.61) | 0.78 (0.75, 0.80) | 0.61 (0.59, 0.63) | 0.70 (0.68, 0.73) |
| Childhood onset disorders (F90–F98) | 0.87 (0.85, 0.89) | 0.94 (0.92, 0.97) | 0.97 (0.95, 0.99) | 0.99 (0.97, 1.01) |
| Any mental disorder (F00–F99) | 0.71 (0.70, 0.71) | 0.77 (0.76, 0.78) | 0.90 (0.89, 0.90) | 0.90 (0.89, 0.91) |

*Note*. HR = hazard ratio, CI = confidence interval. Model 1 adjusted for birth year, educational level, and infertility-related genitourinary; Model 2 additionally adjusted for cohabitation/marriage.

**Table S3.** Rate ratios (95% confidence intervals) of the association between mental disorders and cohabitation.

|  | **Men** | **Women** |
| --- | --- | --- |
| **Diagnosis** | RR (95% CI) | RR (95% CI) |
| Substance use disorders (F10–F19) | 0.63 (0.62, 0.64) | 0.81 (0.80, 0.82) |
| Psychotic disorders (F20–F29) | 0.35 (0.34, 0.36) | 0.56 (0.55, 0.58) |
| Schizophrenia (F20) | 0.20 (0.18, 0.21) | 0.37 (0.35, 0.40) |
| Mood disorders (F30–F39) | 0.56 (0.55, 0.57) | 0.76 (0.76, 0.77) |
| Bipolar disorder (F30–F31) | 0.53 (0.50, 0.55) | 0.69 (0.67, 0.71) |
| Depression (F32–F34) | 0.59 (0.59, 0.60) | 0.80 (0.79, 0.80) |
| Anxiety disorders (F40–F48) | 0.60 (0.59, 0.61) | 0.73 (0.73, 0.74) |
| Behavioural syndromes with physical factors (F50–F59) | 0.56 (0.55, 0.57) | 0.77 (0.76, 0.78) |
| Eating disorders (F50) | 0.63 (0.59, 0.68) | 0.87 (0.86, 0.88) |
| Sleep disorders (F51) | 0.67 (0.65, 0.70) | 0.72 (0.70, 0.74) |
| Personality disorders (F60–F69) | 0.49 (0.48, 0.50) | 0.58 (0.57, 0.59) |
| Intellectual disabilities (F70–F79) | 0.20 (0.19, 0.22) | 0.25 (0.24, 0.27) |
| Developmental disorders (F80–F89) | 0.61 (0.60, 0.62) | 0.68 (0.67, 0.69) |
| Childhood onset disorders (F90–F98) | 0.77 (0.76, 0.78) | 0.84 (0.84, 0.85) |
| Any mental disorder (F00–F99) | 0.65 (0.64, 0.65) | 0.78 (0.78, 0.78) |

*Note*. RR = rate ratio, CI = confidence interval.

**Table S4.** Age-specific hazard ratios (95% confidence intervals) of the association between mental disorders and having a first child.

|  | **Men** | | | **Women** | | |
| --- | --- | --- | --- | --- | --- | --- |
|  | **25** | **30** | **35** | **25** | **30** | **35** |
| **Diagnosis** | HR (95% CI) | HR (95% CI) | HR (95% CI) | HR (95% CI) | HR (95% CI) | HR (95% CI) |
| Substance use disorders (F10–F19) | 1.19 (1.15, 1.24) | 0.91 (0.88, 0.93) | 0.78 (0.76, 0.79) | 1.23 (1.20, 1.27) | 1.02 (1.00, 1.05) | 0.93 (0.91, 0.95) |
| Psychotic disorders (F20–F29) | 0.64 (0.58, 0.70) | 0.45 (0.42, 0.48) | 0.38 (0.36, 0.40) | 0.61 (0.57, 0.66) | 0.50 (0.48, 0.53) | 0.44 (0.43, 0.47) |
| Schizophrenia (F20) | 0.33 (0.26, 0.43) | 0.22 (0.19, 0.26) | 0.18 (0.16, 0.20) | 0.33 (0.27, 0.40) | 0.27 (0.24, 0.31) | 0.24 (0.21, 0.27) |
| Mood disorders (F30–F39) | 0.93 (0.89, 0.96) | 0.71 (0.69, 0.73) | 0.63 (0.62, 0.64) | 1.03 (1.01, 1.05) | 0.85 (0.84, 0.86) | 0.80 (0.79, 0.81) |
| Bipolar disorder (F30–F31) | 0.94 (0.81, 1.08) | 0.61 (0.55, 0.66) | 0.57 (0.53, 0.61) | 0.75 (0.69, 0.81) | 0.64 (0.61, 0.68) | 0.59 (0.56, 0.62) |
| Depression (F32–F34) | 0.92 (0.88, 0.96) | 0.71 (0.69, 0.73) | 0.63 (0.62, 0.65) | 1.04 (1.01, 1.06) | 0.87 (0.86, 0.89) | 0.82 (0.80, 0.83) |
| Anxiety disorders (F40–F48) | 1.05 (1.02, 1.09) | 0.86 (0.84, 0.88) | 0.78 (0.76, 0.79) | 1.07 (1.05, 1.09) | 0.94 (0.93, 0.95) | 0.90 (0.89, 0.91) |
| Behavioural syndromes with physical factors (F50–F59) | 0.90 (0.85, 0.96) | 0.78 (0.74, 0.81) | 0.71 (0.69, 0.73) | 0.94 (0.91, 0.97) | 0.91 (0.89, 0.93) | 0.89 (0.88, 0.91) |
| Eating disorders (F50) | 0.57 (0.44, 0.74) | 0.64 (0.54, 0.75) | 0.61 (0.53, 0.71) | 0.84 (0.80, 0.88) | 0.84 (0.82, 0.87) | 0.84 (0.81, 0.86) |
| Sleep disorders (F51) | 0.92 (0.82, 1.03) | 0.80 (0.74, 0.86) | 0.75 (0.70, 0.80) | 1.00 (0.92, 1.09) | 0.89 (0.83, 0.95) | 0.85 (0.80, 0.91) |
| Personality disorders (F60–F69) | 0.99 (0.92, 1.07) | 0.71 (0.67, 0.75) | 0.59 (0.57, 0.62) | 0.79 (0.75, 0.83) | 0.61 (0.59, 0.64) | 0.55 (0.54, 0.57) |
| Intellectual disabilities (F70–F79) | 0.45 (0.38, 0.53) | 0.31 (0.27, 0.36) | 0.25 (0.22, 0.28) | 0.30 (0.26, 0.34) | 0.21 (0.19, 0.24) | 0.17 (0.15, 0.19) |
| Developmental disorders (F80–F89) | 1.03 (0.98, 1.07) | 0.85 (0.82, 0.88) | 0.79 (0.77, 0.82) | 0.91 (0.87, 0.95) | 0.76 (0.74, 0.79) | 0.71 (0.69, 0.74) |
| Childhood onset disorders (F90–F98) | 1.24 (1.20, 1.28) | 1.04 (1.01, 1.07) | 0.96 (0.93, 0.98) | 1.19 (1.16, 1.22) | 1.06 (1.03, 1.08) | 1.00 (0.98, 1.02) |
| Any mental disorder (F00–F99) | 1.09 (1.07, 1.11) | 0.87 (0.85, 0.88) | 0.78 (0.77, 0.79) | 1.11 (1.10, 1.13) | 0.96 (0.95, 0.97) | 0.91 (0.90, 0.92) |

*Note*. HR = hazard ratio, CI = confidence interval. Models were adjusted for birth year, educational level, and infertility-related genitourinary, and cohabitation/marriage.

**Table S5.** Hazard ratios (95% confidence intervals) of the association between mental disorders and having a first child of the sibling analysis and corresponding model without family stratification.

|  | **Sibling analysis** | **Corresponding analysis** |
| --- | --- | --- |
| **Diagnosis** | HR (95% CI) | HR (95% CI) |
| Substance use disorders (F10–F19) | 0.46 (0.44, 0.47) | 0.52 (0.51, 0.53) |
| Psychotic disorders (F20–F29) | 0.26 (0.25, 0.28) | 0.28 (0.27, 0.29) |
| Schizophrenia (F20) | 0.13 (0.11, 0.15) | 0.14 (0.13, 0.15) |
| Mood disorders (F30–F39) | 0.42 (0.42, 0.43) | 0.45 (0.45, 0.46) |
| Bipolar disorder (F30–F31) | 0.33 (0.31, 0.36) | 0.36 (0.35, 0.37) |
| Depression (F32–F34) | 0.50 (0.48, 0.51) | 0.51 (0.50, 0.52) |
| Anxiety disorders (F40–F48) | 0.41 (0.40, 0.42) | 0.46 (0.45, 0.46) |
| Behavioural syndromes with physical factors (F50–F59) | 0.43 (0.42, 0.45) | 0.45 (0.44, 0.45) |
| Eating disorders (F50) | 0.72 (0.68, 0.76) | 0.66 (0.65, 0.68) |
| Sleep disorders (F51) | 0.48 (0.44, 0.52) | 0.46 (0.44, 0.49) |
| Personality disorders (F60–F69) | 0.34 (0.32, 0.35) | 0.35 (0.34, 0.36) |
| Intellectual disabilities (F70–F79) | 0.13 (0.11, 0.15) | 0.15 (0.14, 0.17) |
| Developmental disorders (F80–F89) | 0.63 (0.60, 0.66) | 0.64 (0.63, 0.66) |
| Childhood onset disorders (F90–F98) | 0.79 (0.77, 0.82) | 0.81 (0.80, 0.82) |
| Any mental disorder (F00–F99) | 0.45 (0.44, 0.46) | 0.51 (0.50, 0.51) |

*Note*. HR = hazard ratio, CI = confidence interval.

**Table S6**. Estimates for cumulative incidence of having a first child among men and women with and without mental disorders.

|  | **Age** | **Exposed** | | **Unexposed** | |
| --- | --- | --- | --- | --- | --- |
| **Diagnosis** |  | **Men** | **Women** | **Men** | **Women** |
| Substance use disorders (F10–F19) |  |  |  |  |  |
|  | 25 | 7.61 (7.34, 7.88) | 20 (19.46, 20.55) | 11.16 (11.08, 11.25) | 20.56 (20.45, 20.66) |
|  | 30 | 18.39 (17.96, 18.83) | 35.79 (35.08, 36.52) | 30.79 (30.65, 30.92) | 44.47 (44.33, 44.61) |
|  | 35 | 28.42 (27.83, 29.02) | 47.97 (47.08, 48.87) | 50.54 (50.37, 50.71) | 63.66 (63.49, 63.82) |
|  | 38 | 32.39 (31.69, 33.11) | 52.51 (51.46, 53.57) | 57.45 (57.25, 57.65) | 69.37 (69.18, 69.55) |
| Psychotic disorders (F20–F29) |  |  |  |  |  |
|  | 25 | 3.05 (2.78, 3.35) | 9.75 (9.16, 10.38) | 11.14 (11.06, 11.22) | 20.71 (20.6, 20.81) |
|  | 30 | 7.7 (7.25, 8.19) | 19.34 (18.49, 20.23) | 30.6 (30.47, 30.73) | 44.58 (44.44, 44.72) |
|  | 35 | 12.67 (12.02, 13.37) | 27.63 (26.53, 28.77) | 50.15 (49.98, 50.32) | 63.74 (63.58, 63.91) |
|  | 38 | 14.74 (13.94, 15.57) | 31.65 (30.31, 33.04) | 57.01 (56.81, 57.21) | 69.45 (69.26, 69.64) |
| Schizophrenia (F20) |  |  |  |  |  |
|  | 25 | 1.22 (0.94, 1.57) | 4.48 (3.69, 5.43) | 11.03 (10.95, 11.11) | 20.6 (20.49, 20.7) |
|  | 30 | 3.35 (2.85, 3.93) | 10.93 (9.64, 12.38) | 30.28 (30.16, 30.41) | 44.32 (44.18, 44.46) |
|  | 35 | 5.6 (4.88, 6.41) | 16.57 (14.86, 18.44) | 49.64 (49.47, 49.81) | 63.36 (63.2, 63.53) |
|  | 38 | 6.39 (5.56, 7.34) | 19.57 (17.5, 21.86) | 56.44 (56.25, 56.64) | 69.07 (68.88, 69.25) |
| Mood disorders (F30–F39) |  |  |  |  |  |
|  | 25 | 5.22 (5.04, 5.41) | 13.75 (13.51, 13.99) | 11.5 (11.41, 11.58) | 21.58 (21.47, 21.69) |
|  | 30 | 13.41 (13.09, 13.73) | 27.75 (27.4, 28.11) | 31.59 (31.45, 31.72) | 46.55 (46.39, 46.7) |
|  | 35 | 23.22 (22.74, 23.71) | 42.01 (41.53, 42.5) | 51.51 (51.34, 51.69) | 65.97 (65.8, 66.14) |
|  | 38 | 27.69 (27.07, 28.31) | 47.99 (47.39, 48.6) | 58.4 (58.2, 58.6) | 71.53 (71.34, 71.72) |
| Bipolar disorder (F30–F31) |  |  |  |  |  |
|  | 25 | 4.11 (3.57, 4.73) | 9.4 (8.75, 10.1) | 11 (10.93, 11.08) | 20.67 (20.57, 20.78) |
|  | 30 | 11.01 (10.07, 12.03) | 22.62 (21.58, 23.7) | 30.21 (30.08, 30.33) | 44.45 (44.31, 44.59) |
|  | 35 | 21.77 (20.29, 23.35) | 34.61 (33.2, 36.05) | 49.46 (49.29, 49.62) | 63.51 (63.34, 63.68) |
|  | 38 | 26.11 (24.26, 28.08) | 39.62 (37.91, 41.38) | 56.2 (56.01, 56.4) | 69.19 (69.01, 69.38) |
| Depression (F32–F34) |  |  |  |  |  |
|  | 25 | 6.12 (5.88, 6.36) | 16.04 (15.74, 16.34) | 11.27 (11.19, 11.35) | 21.03 (20.92, 21.14) |
|  | 30 | 14.74 (14.36, 15.14) | 30.74 (30.33, 31.16) | 31.04 (30.91, 31.17) | 45.6 (45.45, 45.75) |
|  | 35 | 24.06 (23.5, 24.63) | 44.42 (43.88, 44.97) | 50.81 (50.64, 50.98) | 65.02 (64.85, 65.2) |
|  | 38 | 28.37 (27.66, 29.09) | 49.87 (49.21, 50.54) | 57.68 (57.48, 57.88) | 70.68 (70.49, 70.87) |
| Anxiety disorders (F40–F48) |  |  |  |  |  |
|  | 25 | 5.83 (5.66, 6.01) | 11.04 (10.85, 11.23) | 11.59 (11.51, 11.68) | 22.51 (22.39, 22.63) |
|  | 30 | 15.93 (15.62, 16.24) | 25.76 (25.45, 26.07) | 31.75 (31.61, 31.89) | 47.7 (47.54, 47.85) |
|  | 35 | 27.95 (27.49, 28.42) | 42.24 (41.8, 42.7) | 51.67 (51.49, 51.84) | 66.79 (66.61, 66.96) |
|  | 38 | 33.59 (33.01, 34.18) | 49.21 (48.64, 49.78) | 58.49 (58.29, 58.7) | 72.15 (71.96, 72.34) |
| Behavioural syndromes with physical factors | |  |  |  |  |
| (F50–F59) | 25 | 3.91 (3.69, 4.15) | 10.04 (9.74, 10.34) | 11.27 (11.19, 11.35) | 21.29 (21.18, 21.4) |
|  | 30 | 11.53 (11.1, 11.98) | 25.39 (24.89, 25.9) | 30.83 (30.7, 30.96) | 45.41 (45.26, 45.56) |
|  | 35 | 22.06 (21.34, 22.81) | 42.18 (41.44, 42.93) | 50.27 (50.1, 50.44) | 64.4 (64.23, 64.56) |
|  | 38 | 27.66 (26.7, 28.65) | 49.21 (48.27, 50.15) | 57.02 (56.82, 57.22) | 69.96 (69.78, 70.15) |
| Eating disorders (F50) |  |  |  |  |  |
|  | 25 | 4.17 (3.22, 5.4) | 12.29 (11.76, 12.83) | 10.97 (10.89, 11.05) | 20.75 (20.64, 20.85) |
|  | 30 | 14.16 (12.1, 16.54) | 30.61 (29.75, 31.48) | 30.09 (29.96, 30.22) | 44.51 (44.37, 44.65) |
|  | 35 | 24.79 (21.49, 28.51) | 47.84 (46.64, 49.04) | 49.28 (49.11, 49.45) | 63.49 (63.33, 63.66) |
|  | 38 | 27.51 (23.74, 31.74) | 54.31 (52.82, 55.82) | 56.01 (55.82, 56.21) | 69.14 (68.96, 69.33) |
| Sleep disorders (F51) |  |  |  |  |  |
|  | 25 | 5.85 (5.23, 6.53) | 11.23 (10.32, 12.21) | 10.99 (10.92, 11.07) | 20.61 (20.5, 20.71) |
|  | 30 | 15.7 (14.6, 16.88) | 23.47 (22.07, 24.93) | 30.17 (30.04, 30.3) | 44.32 (44.18, 44.47) |
|  | 35 | 26.69 (25.04, 28.43) | 37.78 (35.72, 39.92) | 49.4 (49.24, 49.57) | 63.31 (63.15, 63.48) |
|  | 38 | 32.75 (30.57, 35.03) | 43.88 (41.29, 46.56) | 56.13 (55.94, 56.33) | 68.99 (68.8, 69.17) |
| Personality disorders (F60–F69) |  |  |  |  |  |
|  | 25 | 5.69 (5.28, 6.12) | 9.49 (9.04, 9.96) | 11.05 (10.98, 11.13) | 20.83 (20.73, 20.94) |
|  | 30 | 13.59 (12.94, 14.27) | 20.47 (19.78, 21.19) | 30.39 (30.26, 30.52) | 44.81 (44.67, 44.95) |
|  | 35 | 21.64 (20.74, 22.57) | 31.08 (30.13, 32.05) | 49.81 (49.64, 49.98) | 63.98 (63.82, 64.15) |
|  | 38 | 25.25 (24.17, 26.37) | 35.59 (34.43, 36.78) | 56.62 (56.43, 56.82) | 69.67 (69.49, 69.86) |
| Intellectual disabilities (F70–F79) |  |  |  |  |  |
|  | 25 | 2.65 (2.23, 3.14) | 5.63 (4.91, 6.45) | 11.02 (10.94, 11.1) | 20.62 (20.52, 20.73) |
|  | 30 | 4.85 (4.23, 5.56) | 8.8 (7.84, 9.88) | 30.24 (30.11, 30.37) | 44.38 (44.24, 44.52) |
|  | 35 | 6.31 (5.52, 7.21) | 11.16 (9.97, 12.5) | 49.53 (49.36, 49.69) | 63.43 (63.27, 63.6) |
|  | 38 | 7.18 (6.21, 8.29) | 11.48 (10.22, 12.9) | 56.29 (56.1, 56.49) | 69.13 (68.95, 69.32) |
| Developmental disorders (F80–F89) |  |  |  |  |  |
|  | 25 | 8.51 (8.17, 8.85) | 16.83 (16.18, 17.51) | 11.06 (10.98, 11.14) | 20.61 (20.51, 20.72) |
|  | 30 | 17.57 (17, 18.15) | 28.23 (27.27, 29.22) | 30.45 (30.32, 30.57) | 44.42 (44.28, 44.57) |
|  | 35 | 23.44 (22.57, 24.33) | 36.34 (34.95, 37.77) | 49.77 (49.6, 49.94) | 63.45 (63.28, 63.61) |
|  | 38 | 25.49 (24.39, 26.64) | 39.05 (37.29, 40.85) | 56.51 (56.32, 56.71) | 69.12 (68.93, 69.3) |
| Childhood onset disorders (F90–F98) |  |  |  |  |  |
|  | 25 | 12.16 (11.79, 12.54) | 24.39 (23.82, 24.96) | 10.89 (10.81, 10.97) | 20.38 (20.28, 20.49) |
|  | 30 | 23.91 (23.34, 24.49) | 38.41 (37.66, 39.17) | 30.27 (30.14, 30.4) | 44.32 (44.17, 44.46) |
|  | 35 | 32.98 (32.16, 33.83) | 48.81 (47.8, 49.83) | 49.7 (49.53, 49.87) | 63.46 (63.29, 63.62) |
|  | 38 | 37.18 (36.05, 38.32) | 52.2 (50.96, 53.46) | 56.45 (56.26, 56.65) | 69.15 (68.97, 69.34) |
| Any mental disorder (F00–F99) |  |  |  |  |  |
|  | 25 | 7.5 (7.36, 7.63) | 14.32 (14.15, 14.49) | 12.05 (11.96, 12.14) | 22.97 (22.84, 23.1) |
|  | 30 | 18.32 (18.1, 18.55) | 30.07 (29.82, 30.33) | 33.43 (33.28, 33.58) | 49.18 (49.02, 49.35) |
|  | 35 | 30.09 (29.76, 30.42) | 46.22 (45.87, 46.56) | 54.27 (54.08, 54.45) | 68.6 (68.42, 68.78) |
|  | 38 | 35.43 (35.01, 35.84) | 52.74 (52.32, 53.17) | 61.24 (61.02, 61.45) | 73.86 (73.66, 74.06) |

**Table S7.** Hazard ratios (95% confidence intervals) of the association between mental disorders and having a first child using only secondary health care data.

|  | **Men (person years=8321084)** | | **Women (person years = 7062198)** | |
| --- | --- | --- | --- | --- |
|  | Model 1 | Model 2 | Model 1 | Model 2 |
| **Diagnosis** | HR (95% CI) | HR (95% CI) | HR (95% CI) | HR (95% CI) |
| Substance use disorders (F10–F19) | 0.73 (0.72, 0.75) | 0.74 (0.73, 0.76) | 0.98 (0.95, 1.00) | 0.92 (0.90, 0.94) |
| Psychotic disorders (F20–F29) | 0.27 (0.26, 0.28) | 0.37 (0.35, 0.39) | 0.40 (0.38, 0.42) | 0.44 (0.42, 0.46) |
| Schizophrenia (F20) | 0.11 (0.09, 0.12) | 0.17 (0.15, 0.19) | 0.20 (0.18, 0.23) | 0.23 (0.21, 0.26) |
| Mood disorders (F30–F39) | 0.57 (0.56, 0.58) | 0.62 (0.60, 0.63) | 0.81 (0.8, 0.82) | 0.80 (0.79, 0.81) |
| Bipolar disorder (F30–F31) | 0.53 (0.49, 0.57) | 0.56 (0.52, 0.60) | 0.59 (0.57, 0.62) | 0.58 (0.56, 0.61) |
| Depression (F32–F34) | 0.57 (0.56, 0.58) | 0.62 (0.60, 0.63) | 0.81 (0.80, 0.83) | 0.81 (0.80, 0.82) |
| Anxiety disorders (F40–F48) | 0.70 (0.69, 0.72) | 0.74 (0.73, 0.76) | 0.87 (0.86, 0.89) | 0.87 (0.86, 0.88) |
| Behavioural syndromes with physical factors (F50–F59) | 0.69 (0.65, 0.72) | 0.73 (0.69, 0.77) | 0.84 (0.81, 0.86) | 0.85 (0.83, 0.87) |
| Eating disorders (F50) | 0.52 (0.45, 0.60) | 0.62 (0.54, 0.72) | 0.82 (0.80, 0.85) | 0.84 (0.82, 0.87) |
| Sleep disorders (F51) | 0.73 (0.69, 0.78) | 0.75 (0.70, 0.80) | 0.86 (0.81, 0.91) | 0.86 (0.81, 0.91) |
| Personality disorders (F60–F69) | 0.51 (0.49, 0.53) | 0.57 (0.55, 0.60) | 0.54 (0.52, 0.56) | 0.55 (0.53, 0.57) |
| Intellectual disabilities (F70–F79) | 0.14 (0.12, 0.15) | 0.24 (0.21, 0.28) | 0.12 (0.11, 0.14) | 0.16 (0.14, 0.18) |
| Developmental disorders (F80–F89) | 0.59 (0.57, 0.61) | 0.78 (0.76, 0.81) | 0.61 (0.59, 0.64) | 0.71 (0.68, 0.73) |
| Childhood onset disorders (F90–F98) | 0.88 (0.86, 0.90) | 0.96 (0.94, 0.98) | 0.99 (0.97, 1.01) | 1.00 (0.98, 1.03) |
| Any mental disorder (F00–F99) | 0.69 (0.68, 0.70) | 0.76 (0.75, 0.77) | 0.86 (0.85, 0.87) | 0.87 (0.86, 0.88) |

*Note*. HR = hazard ratio, CI = confidence interval. Model 1 adjusted for birth year, educational level, and infertility-related genitourinary; Model 2 additionally adjusted for cohabitation/marriage.


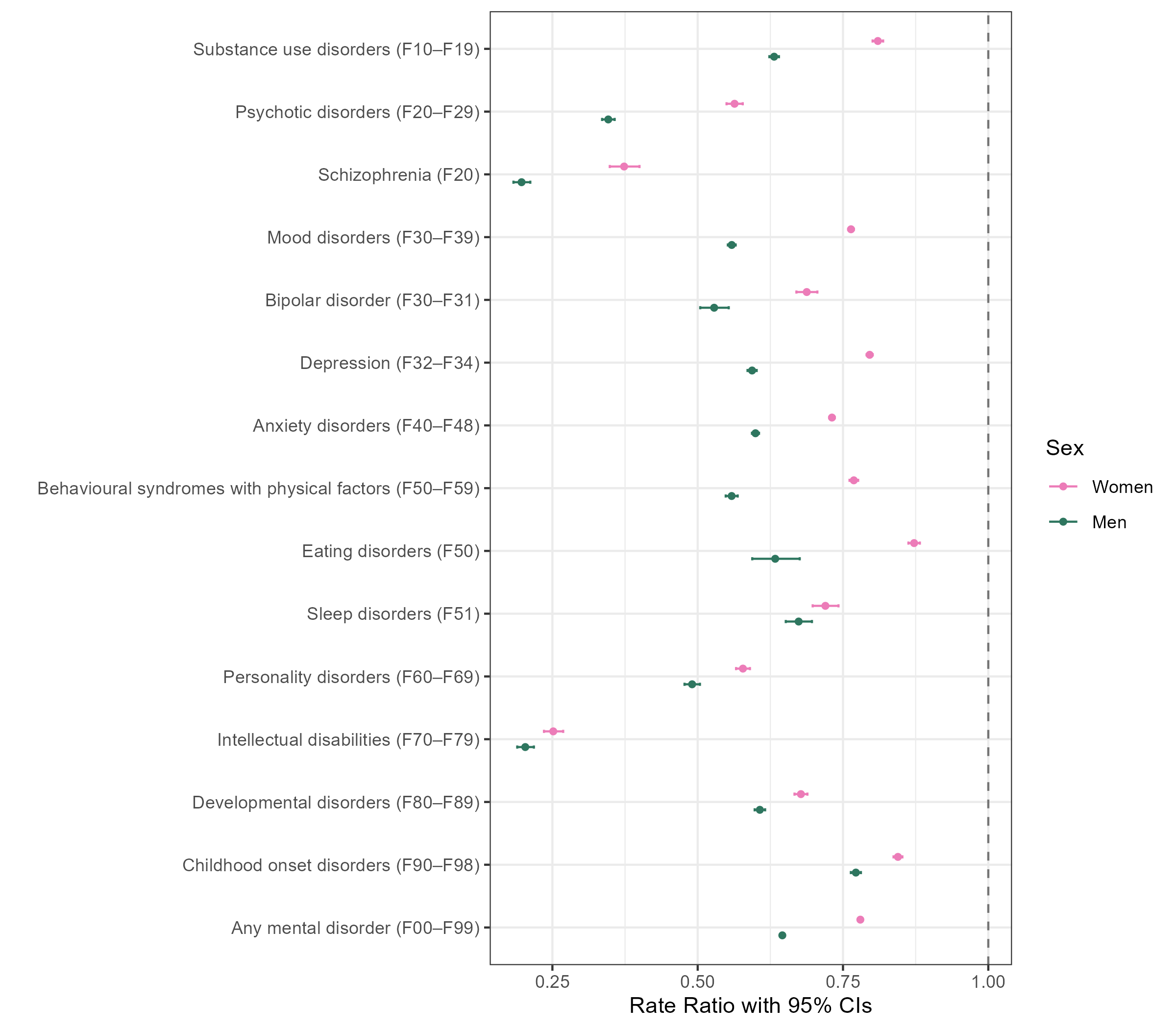


**Figure S1**. Rate ratios (95% confidence intervals) of the association between mental disorders and cohabitation. Estimates are shown in Table S3.


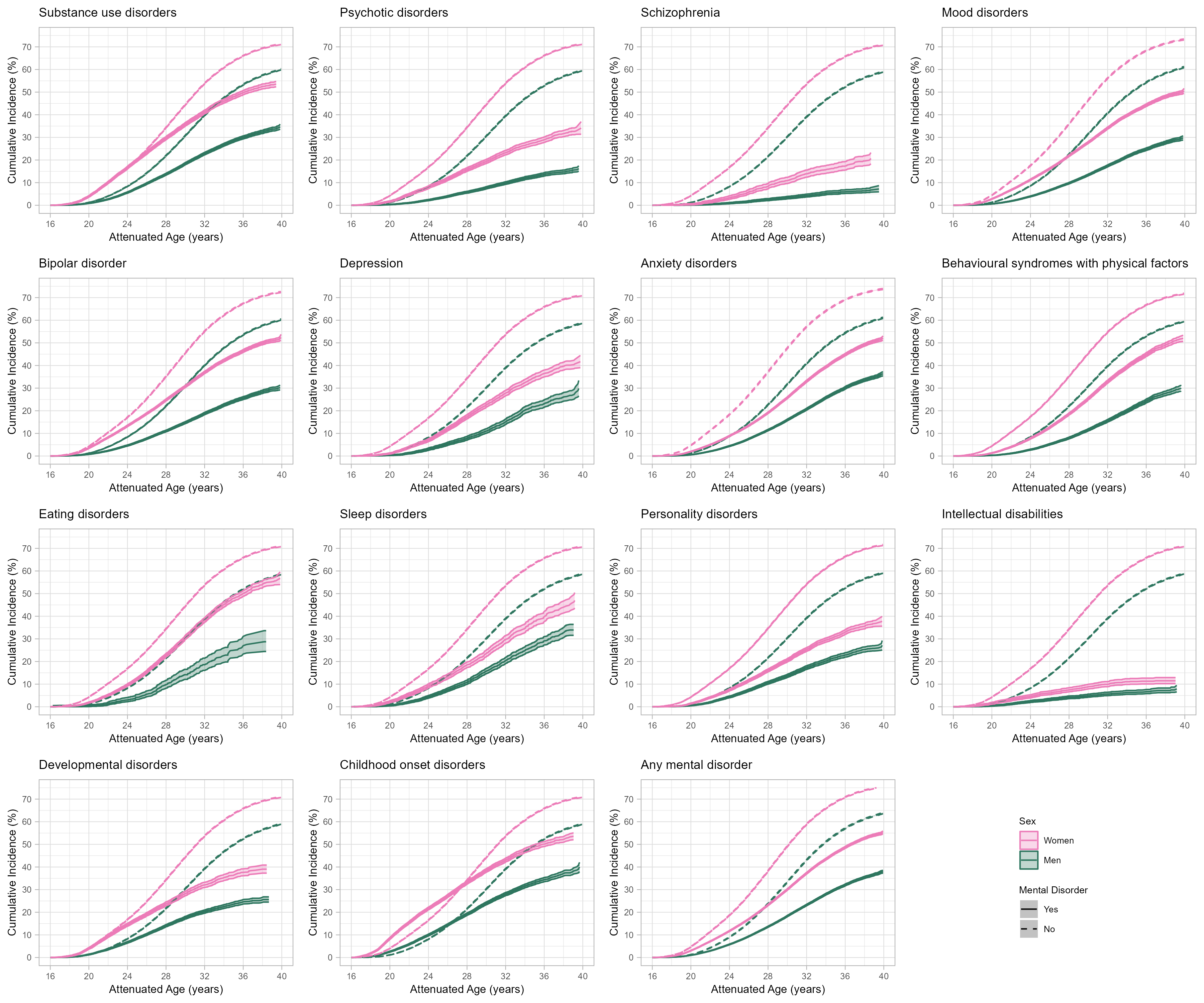


**Figure S2.** Cumulative incidence of having a first child among men and women with and without mental disorders. Estimates are shown in Table S6.


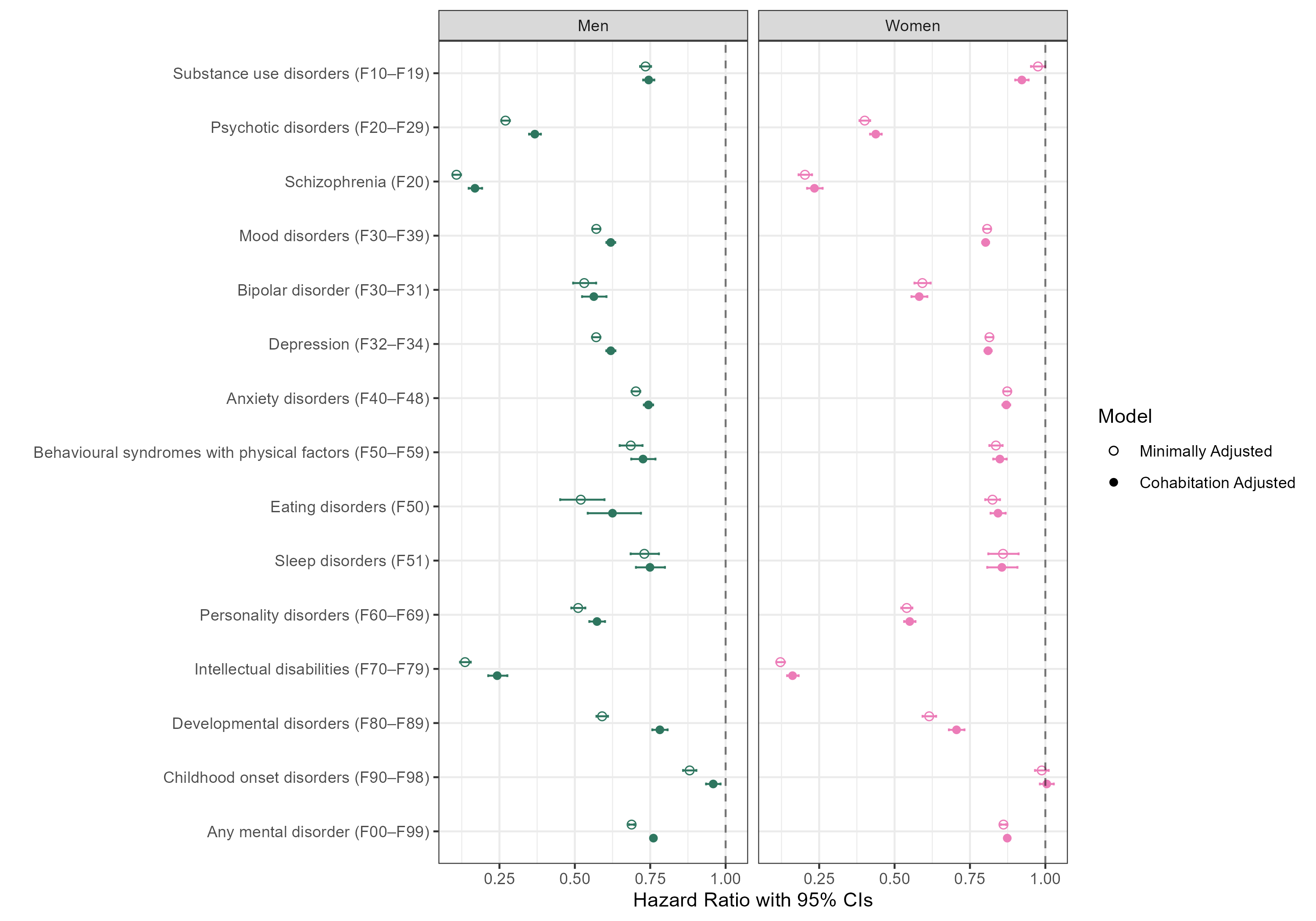


**Figure S3.** Hazard ratios (95% confidence intervals) of the association between mental disorders and having a first child using only secondary health care data. Estimates are shown in Table S7.
